# Temporalis Muscle Thickness as a Prognostic Marker in Acute Ischemic Stroke: A Systematic Review

**DOI:** 10.3390/jcm15187240

**Published:** 2026-09-17

**Authors:** Holden Husbands, Ahmed Hussan, William J. P. Ertl, Hanyu Qiu, Jack E. Stanfield, Kishore Balasubramanian, Hakeem J. Shakir

**Affiliations:** Department of Neurosurgery, University of Oklahoma College of Medicine, Oklahoma City, OK 73104, USA; holden-husbands@ou.edu (H.H.); ahmed-hussan@ou.edu (A.H.); will-ertl@ou.edu (W.J.P.E.); hanyu-qiu@ou.edu (H.Q.); jack-stanfield@ou.edu (J.E.S.); kishore-balasubramanian@ou.edu (K.B.)

**Keywords:** ischemic stroke, sarcopenia, temporalis muscle, prognosis, neuroimaging

## Abstract

**Background**: Temporalis muscle thickness (TMT) measured on routine neuroimaging has emerged as a radiological marker of sarcopenia, but its prognostic value in acute ischemic stroke is not well defined. This systematic review examined whether TMT is associated with functional status, swallowing outcomes, and mortality after acute ischemic stroke. **Methods**: A PRISMA-guided systematic review identified observational studies (2000–2025) reporting TMT and at least one clinical outcome in adults with acute ischemic stroke. PubMed, MEDLINE, Embase, Web of Science, and Scopus were searched. Two reviewers independently screened records and extracted data. Risk of bias was assessed using ROBINS-I and the Newcastle–Ottawa Scale, with justification relative to prognostic-factor tools. Due to heterogeneity in TMT definitions and outcome measures, findings were synthesized narratively. **Results**: Four cohorts, including 1361 patients, met the inclusion criteria. Mean age was 69.7 years, and 53.9% were male. Study-level mean TMT ranged from 5.2 to 6.35 mm; among the 704 patients in cohorts that reported TMT-defined sarcopenia status, prevalence was 51.8% (365/704). Across studies, lower TMT or TMT-defined sarcopenia was associated with poorer functional outcomes and higher mortality, independent of age and stroke severity. In one thrombectomy cohort, each 1-mm increase in TMT increased the odds of functional independence (adjusted OR 1.14, 95% CI 1.03–1.27). In contrast, each 1-mm increase reduced 90-day mortality (adjusted OR 0.71, 95% CI 0.54–0.93). Lower TMT also predicted prolonged nasogastric tube dependence. **Conclusions**: TMT is a simple CT- or MRI-based measure that is consistently associated with disability, dysphagia, and survival after acute ischemic stroke. Current evidence is observational and supports further prospective validation and measurement standardization rather than immediate routine decision-making use.

## 1. Introduction

Stroke is a leading cause of disability and mortality globally. Acute disruption of cerebral blood flow causes neuronal death, resulting in permanent neurological damage and disabilities. Timely diagnosis and treatment are crucial determinants of patient outcomes. Post-stroke recovery is influenced by multiple factors, including treatment timing, stroke severity, and baseline patient characteristics [1]. Sarcopenia, present in 42–46% of stroke patients and defined as progressive loss of skeletal muscle mass, strength, and function, can independently predict poor functional outcomes, increased mortality, and prolonged hospitalization [2,3,4]. Its diagnosis traditionally relies on dual-energy X-ray absorptiometry (DXA), bioelectrical impedance analysis (BIA), grip strength, and physical performance testing. These methods are often impractical in acute stroke patients who may be immobilized or cognitively impaired [5].

Temporalis muscle thickness (TMT) has emerged as a promising alternative indicator of sarcopenia in the context of neurological injury. In acute stroke, TMT can be measured from routine cranial MRI or CT at the level of the orbital roof, perpendicular to the long axis of the temporalis muscle, allowing for immediate profiling without the need for additional imaging or delays [6]. TMT may serve as a systemic indicator of nutritional and metabolic health, associated with levels of frailty, hormonal status, and muscle function. Beyond stroke, sarcopenia predicts adverse post-surgical outcomes in multiple clinical populations, including prolonged recovery time, increased complications, extended hospitalization, and higher mortality [7,8].

Serum biomarkers such as glial fibrillary acidic protein, neurofilament light chain, neuron-specific enolase, and S100B reflect acute neuronal or glial injury and can predict short-term outcomes, but they capture acute damage rather than baseline physiological reserve. In contrast, TMT reflects underlying muscle status and may provide complementary prognostic information [9].

TMT has also been linked to clinical outcomes in other neurological populations, including aneurysmal subarachnoid hemorrhage, supporting biological plausibility for cranial muscle measures as markers of reserve, although those populations were outside the eligibility criteria of the present AIS-focused review [10]. Measurements can be performed rapidly without additional sequences or contrast or disrupting acute stroke workflows, while allowing muscle mass assessment in critically ill patients unable to undergo physical examinations [11].

To date, no systematic review has investigated the correlation between TMT and sarcopenia and its prognostic value specifically in acute ischemic stroke. This systematic review examines whether TMT is associated with key clinical outcomes, including functional status, swallowing ability, and mortality, in adults with acute ischemic stroke.

## 2. Methods

### 2.1. Literature Review

**Figure 1:**
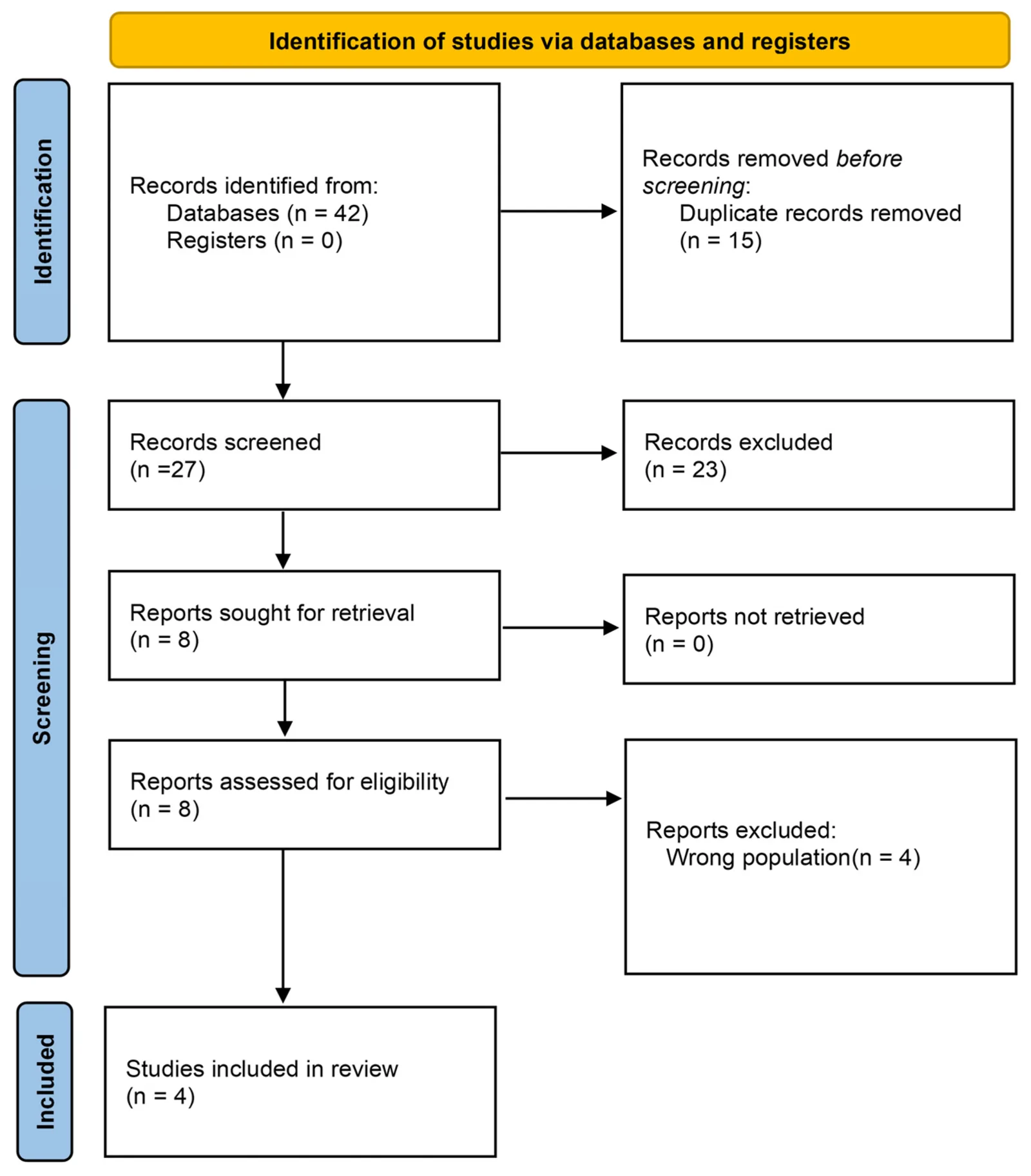
PRISMA flow diagram showing the identification, screening, eligibility, and inclusion of studies in this review. Records were identified through database searching in PubMed (n = 24), Web of Science (n = 5), Embase (n = 7), and Scopus (n = 6), for a total of 42 records. After removal of 15 duplicates, 27 records underwent title and abstract screening, of which 19 were excluded. Eight reports were sought for retrieval; zero were not retrieved. Eight full-text reports were assessed for eligibility; four were excluded because the population was not restricted to acute ischemic stroke (including chronic subdural hematoma and mixed cerebrovascular cohorts), and four studies were included in the final review.

A systematic review was conducted following PRISMA guidelines to identify studies evaluating the relationship between temporalis muscle thickness (TMT) and acute ischemic stroke [12]. PubMed, MEDLINE (Ovid), Embase, Web of Science, and Scopus were searched from 2000 to December 2025 using the terms “temporalis muscle thickness”, “sarcopenia”, “stroke”, and “outcome”. A PRISMA 2020 flow diagram summarizing study selection, including records identified from each database, is provided in Figure 1. The review was not prospectively registered in PROSPERO. Lack of preregistration is acknowledged as a methodological limitation. MEDLINE was searched via Ovid, and PubMed was searched separately to capture ahead-of-print and in-process records not yet indexed in MEDLINE. Full database-specific search strategies, including Boolean operators and any limits, are provided in Supplementary Table S1. PRSMA checklist is provided in Supplementary Table S2.

### 2.2. Study Selection

Studies were included if they (1) enrolled adults with acute ischemic stroke, and (2) reported temporalis muscle thickness measurement together with at least one clinical outcome and follow-up data. Studies were excluded if (1) patients did not have acute ischemic stroke (including hemorrhagic stroke alone, mixed cerebrovascular disease without an ischemic stroke–specific analysis, or chronic subdural hematoma), (2) TMT was not measured, or (3) outcome or follow-up data were not reported. This narrower eligibility criterion was applied to ensure that prognostic inferences are not extrapolated to ICH, SAH, or non-stroke neurosurgical populations without direct supporting data.

### 2.3. Data Extraction

All articles were imported into Rayyan, a web-based screening tool [13]. Two independent reviewers (H.H. and A.H.) screened titles and abstracts, then assessed full texts of articles meeting the inclusion criteria. A third reviewer (K.B.) resolved disagreements.

### 2.4. Data Synthesis and Quality Assessment

The relationship between TMT and cerebrovascular disease was synthesized across included studies. Emphasis was placed on the consistency and strength of these associations. All studies meeting the predefined inclusion criteria were included; no study was excluded solely on the basis of risk-of-bias rating.

### 2.5. Risk of Bias/Quality of Evidence

**Figure 2:**
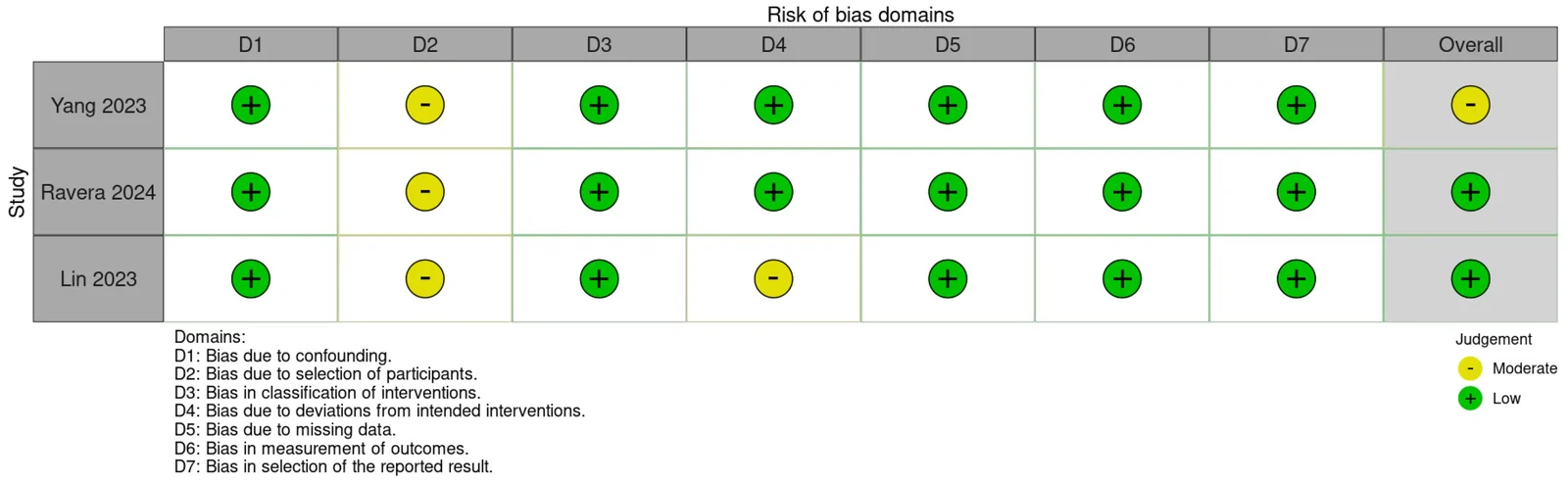
ROBINS-I domain-level risk-of-bias assessment for the three comparative acute ischemic stroke cohorts (Lin 2023 [16], Yang 2023 [17], and Ravera 2024 [18]).

**Figure 3:**
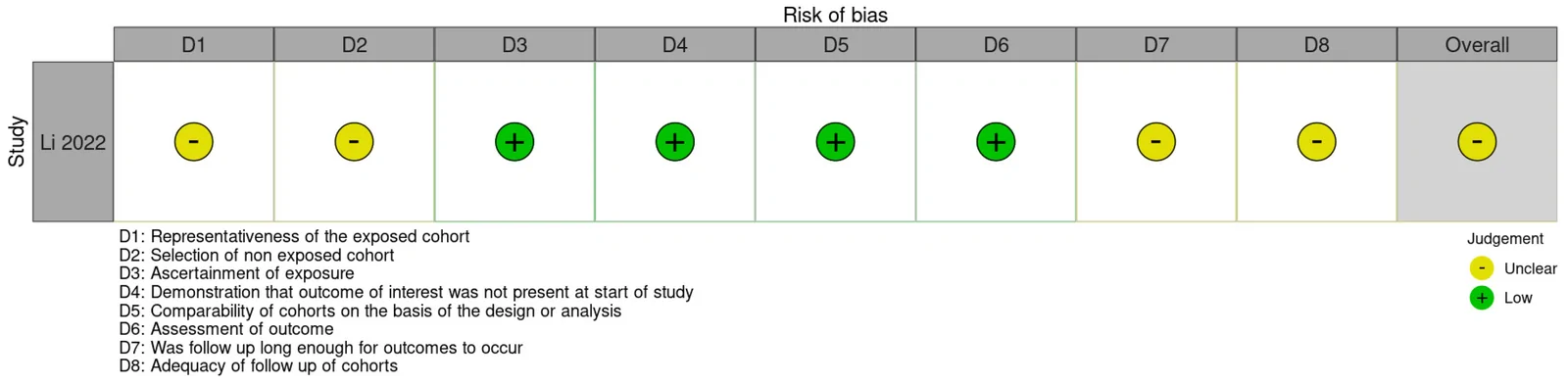
Newcastle–Ottawa Scale domain-level risk-of-bias assessment for the Li 2022 [19] acute ischemic stroke cohort.

Risk of bias was assessed by two reviewers (H.H. and W.E.). Because the included studies primarily evaluated prognostic associations of TMT rather than effects of an assigned intervention, a tool developed specifically for prognostic-factor studies (e.g., QUIPS) would also be appropriate. We nonetheless used ROBINS-I for the three non-randomized comparative cohorts that analyzed TMT in relation to clinical outcomes after reperfusion therapies (Yang, Ravera, Lin) and the Newcastle–Ottawa Scale (NOS) for the remaining single-arm/survival cohort (Li), for two reasons: (1) these instruments are widely used for observational stroke cohorts and facilitate domain-level comparison with prior cerebrovascular reviews; and (2) they were applied consistently within comparable study designs (comparative outcome cohorts vs. single prognostic cohort). We acknowledge that ROBINS-I was developed for non-randomized intervention studies and that residual prognostic-specific biases (e.g., study participation, prognostic factor measurement) may be better captured by QUIPS; this choice is therefore a methodological limitation. Domain-level judgments are summarized in Figure 2 (ROBINS-I; Yang, Ravera, and Lin) and Figure 3 (NOS; Li) [14,15].

### 2.6. Statistical Analysis

A narrative synthesis was performed because the existing data were too heterogeneous to be suitable for meta-analysis.

## 3. Results

### 3.1. Literature Search and Study Characteristics

Four articles met the inclusion criteria after the systematic literature search [16,17,18,19]. A total of 1361 patients were included across four observational acute ischemic stroke cohorts. An overview of studies is provided in Table 1. The weighted mean age across studies was 69.7 years, with a sex distribution of 53.9% male and 46.1% female. All four cohorts enrolled patients with acute ischemic stroke, including large-vessel occlusion and endovascular therapy populations. Study-level mean temporalis muscle thickness ranged from 5.2 to 6.35 mm (approximate sample-size–weighted mean of study means, 5.9 mm). Among the 704 patients in the three cohorts that reported TMT-defined sarcopenia status, sarcopenia prevalence was 51.8% (365/704), though definitions varied: some studies used sex-specific thresholds while others analyzed TMT as a continuous variable or by quartile. A summary of patient demographics and interventions among patients with available data is provided in Table 2.

### 3.2. TMT and Functional Outcomes

Across cohorts reporting dichotomized mRS outcomes, sarcopenic or low-TMT patients had markedly lower rates of functional independence (mRS 0–2) and higher rates of severe disability or death (mRS 5–6) than non-sarcopenic patients. Detailed sarcopenia-stratified mRS distributions were not formally pooled because of heterogeneity in reporting, but are provided by the study in Table 3.

In the largest thrombectomy cohort, Lin et al. (n = 657) reported a graded association between sex-specific TMT quartiles and 90-day functional outcome. Ordinal logistic regression showed that higher TMT quartiles were associated with better 90-day mRS scores (Ptrend = 0.047), and patients in Q2–Q4 had higher odds of achieving functional independence (mRS 0–2) compared with Q1 (adjusted OR 2.23, 95% CI 1.35–3.67; 1.94, 95% CI 1.18–3.19; and 2.31, 95% CI 1.39–3.84; Ptrend = 0.004), after adjustment for age, sex, NIHSS, premorbid mRS, and recanalization status. When modeled continuously, each 1-mm increase in TMT independently increased the odds of functional independence (adjusted OR 1.14, 95% CI 1.03–1.27, *p* ≈ 0.016).

### 3.3. TMT and Mortality

Ravera et al. (n = 291) reported that higher continuous TMT was independently associated with reduced 90-day mortality (adjusted OR for death 0.708 per mm, 95% CI 0.538–0.930, *p* = 0.013) after adjusting for age, diabetes, stroke severity, and intracranial hemorrhage. Using sex-specific TMT cut-offs, 67.6% of their cohort met criteria for sarcopenia; these patients had higher 90-day mortality (26.9% vs. 6.4%, *p* < 0.001) and were less likely to achieve mRS 0–3 (56.9% vs. 75.5%, *p* = 0.002) than non-sarcopenic patients.

Li et al. reported that patients with lower TMT-defined sarcopenia had shorter survival, with a mean survival of 36 months in the low-TMT group versus 49 months in the high-TMT group, and low TMT remaining independently associated with mortality in multivariable models.

### 3.4. TMT and Dysphagia

Yang et al. (n = 148 EVT patients) found that lower TMT and masseter muscle thickness were associated with persistent nasogastric tube dependence at both 4 and 12 weeks. After adjustment for age, sex, body mass index, albumin, and baseline NIHSS, logistic regression across TMT quartiles showed a steep decline in the odds of NG tube retention with increasing TMT (e.g., at 4 weeks, adjusted OR for NG retention vs. Q1: Q2 0.04, 95% CI 0.01–0.35, *p* = 0.003; Q3 0.001, *p* < 0.001; Q4 0.001, *p* < 0.001; Ptrend < 0.001), with similar patterns at 12 weeks. Receiver-operating-characteristic analyses yielded areas under the curve of 0.94 for NG retention at 4 weeks and 0.86 at 12 weeks, with optimal TMT cut-offs around 5.8 mm and 4.7 mm, respectively.

### 3.5. TMT and Systemic Muscle Mass

None of the four included acute ischemic stroke cohorts reported a direct comparison of TMT against whole-body muscle mass by DXA or bioimpedance within the same AIS sample. Related work in broader cerebrovascular and aging populations has reported moderate to strong correlations between cranial masticatory muscle measures and systemic muscle mass, supporting biological plausibility that TMT reflects generalized sarcopenia rather than a purely local finding [20]. This remains an important evidence gap for AIS-specific validation.

### 3.6. Risk of Bias

ROBINS-I assessment indicated predominantly low risk across most domains for Lin 2023 [16] and Ravera 2024 [18], with Yang 2023 [17] judged low risk in confounding, classification, and outcome measurement but at moderate risk in selection of participants. NOS assessment for Li 2022 [19] indicated generally low to unclear risk across representativeness, ascertainment, and outcome domains.

## 4. Discussion

### 4.1. Summary of Key Findings

This systematic review of four acute ischemic stroke studies including 1361 patients suggests that temporalis muscle thickness (TMT) may serve as a surrogate marker for sarcopenia and is independently associated with functional status, swallowing ability, and mortality after ischemic stroke. Across cohorts that reported sarcopenia-stratified functional outcomes, low-TMT or sarcopenic patients generally had lower rates of functional independence and higher rates of severe disability or death than non-sarcopenic patients; these study-level contrasts are summarized descriptively and should not be interpreted as a formal pooled meta-analytic estimate given heterogeneity in thresholds and reporting. For dysphagia, TMT demonstrated capacity to determine prolonged nasogastric tube dependence (AUC 0.94 at 4 weeks) in one EVT cohort. TMT was also associated with 90-day mortality and longer-term survival. These associations often remained significant after multivariable adjustment for age, stroke severity, and comorbidities, suggesting TMT may be an independent prognostic marker within AIS populations. However, all included evidence is observational, and no included study demonstrated that TMT-guided clinical decisions improve outcomes.

### 4.2. Functional Outcomes and Mortality

Across cohorts, patients with sarcopenia or low TMT had substantially lower chances of regaining independence and higher risks of severe disability or death than those with preserved TMT, indicating that cranial muscle thickness captures clinically meaningful differences in biological reserve.

Although the effect per millimeter of TMT is statistically modest, its impact across clinically relevant groups is clinically meaningful. In the largest thrombectomy cohort, each 1-mm increase in TMT was associated with a 14% increase in the odds of functional independence (adjusted OR 1.14, 95% CI 1.03–1.27), suggesting a graded association between cranial muscle thickness and recovery, in line with the stepwise improvement seen across TMT quartiles in the same cohort. Similarly, Ravera et al. reported that each 1 mm increase in TMT reduced 90-day mortality by approximately 30% (adjusted OR 0.71, 95% CI 0.54–0.93). This translated to a greater than fourfold difference in crude mortality between sarcopenic and non-sarcopenic patients (26.9% vs. 6.4%, *p* < 0.001), emphasizing the clinical magnitude of TMT-defined frailty. In Li et al., low-TMT sarcopenia was associated with shorter survival (mean 36 vs. 49 months) and remained independently associated with mortality (hazard ratio, 3.54; 95% confidence interval, 1.46–8.58; *p* < 0.01).

### 4.3. Dysphagia and Systemic Sarcopenia

Beyond functional disability and mortality, TMT was correlated with dysphagia-related outcomes. Yang et al. found that lower TMT was associated with persistent nasogastric tube dependence, with ROC-AUC values of 0.94 for NG retention at 4 weeks and 0.86 at 12 weeks. Masseter muscle thickness also showed predictive ability for post-stroke dysphagia in this cohort, suggesting that multiple masticatory muscles may serve as complementary indicators. Importantly, these cohorts operationalized sarcopenia as radiographically defined low TMT rather than by consensus EWGSOP/AWGS criteria, which require combined evidence of low muscle mass, strength and performance. TMT should therefore be viewed as a screening surrogate for systemic sarcopenia rather than a definitive diagnostic standard.

As a potential cranial indicator of sarcopenia, masseter cross-sectional area (CSA) was found to independently predict sarcopenia after adjustment, while temporalis CSA was not [21]. However, the greater vulnerability of the temporalis to age-related atrophy may make it a more sensitive marker for detecting early or progressive sarcopenia. Both the temporalis and masseter undergo progressive age-related atrophy, with the temporalis decreasing by approximately 0.15 mm/year and the masseter by approximately 0.12 mm/year [22].

Together, these findings support TMT as a candidate cranial marker of generalized muscle depletion rather than a purely local phenomenon. Although AIS-specific DXA/bioimpedance validation was not available among included studies, related cerebrovascular cohorts have reported correlations between TMT and systemic muscle mass, suggesting that thin temporalis musculature may reflect general muscle depletion [20]. These findings suggest that while the prognostic impact per millimeter of TMT is incremental, the difference between low and preserved TMT profiles may represent a meaningful difference in biological reserve.

### 4.4. Implications for Practice and Imaging-Informed Rehabilitation

The consistent association between TMT and ischemic stroke outcomes across multiple cohorts suggests potential clinical utility for early risk stratification. In the included studies, TMT predicted functional independence, mortality, and dysphagia severity—outcomes that directly inform treatment planning. Importantly, TMT measurement requires no additional imaging beyond standard admission head CT or MRI already obtained for stroke evaluation, making it a practical candidate marker for early risk stratification in acute stroke workflows [23]. At the same time, imaging is increasingly being incorporated into individualized post-stroke muscle assessment and treatment planning beyond pure prognostic measurement. Recent work has described detailed ultrasound-guided approaches for identifying individual spastic muscles, their neurovascular relationships, and optimal botulinum toxin injection targets in post-stroke rehabilitation, illustrating how muscle imaging can contribute to precision treatment rather than serving only as a diagnostic or prognostic measurement [24]. Positioning TMT within this broader evolution toward imaging-informed rehabilitation strategies helps clarify both its promise and its current limits: TMT is best viewed as a promising, readily extractable prognostic marker that still requires prospective validation and standardized measurement before routine decision-making use.

Sarcopenia imposes a substantial economic burden on healthcare systems and patients. While the full economic burden of sarcopenia remains incompletely characterized, the most comprehensive US estimate, now over two decades old, reported direct healthcare costs of sarcopenia totaling $18.5 billion (year 2000 dollars) [25]. Given the aging population and rising healthcare costs, the current burden is likely substantially higher. More recent European data demonstrate that sarcopenia increases hospitalization costs by 34–58% per admission, with combined frailty and sarcopenia increasing costs by 46–56% per patient per year compared to non-frail, non-sarcopenic individuals [26]. Given these costs, early identification of at-risk patients through accessible screening methods like TMT could allow for upstream interventions that reduce healthcare utilization.

The dose–response relationships observed—where each 1 mm increase in TMT was associated with 14% higher odds of functional independence and approximately 30% lower odds of 90-day mortality in individual cohorts—suggest that early identification of low-TMT patients could, in future prospective work, help triage rehabilitation intensity and nutritional support. Severe sarcopenia generally requires multi-dimensional strategies combining resistance exercise with nutritional supplementation, and in acute stroke specifically, high energy intake paired with intensive rehabilitation has been associated with prevention of hospital-associated sarcopenia and improved functional independence [27,28]. The strong predictive capacity for dysphagia is particularly relevant, as early identification of patients at risk for prolonged nasogastric tube dependence may guide swallowing therapy and feeding decisions. These implications remain hypothesis-generating until interventional studies test whether TMT-triggered pathways change outcomes.

Though the studies in this review used varying definitions and thresholds, recently proposed age- and sex-adjusted TMT reference values may provide a standardized framework for future radiological sarcopenia assessment [29]. The results of this systematic review support TMT as a clinically meaningful sarcopenia surrogate in acute ischemic stroke, with consistent associations between lower TMT and adverse outcomes across the included AIS cohorts, despite heterogeneity in sarcopenia definitions.

### 4.5. Limitations

This systematic review is constrained by variation in reported mRS outcomes and by differing definitions of key parameters. Definitions of sarcopenia varied widely across studies, with some using sex-specific TMT thresholds and others analyzing TMT as a continuous variable or by quartiles. Functional outcomes were also heterogeneous; while mRS was the most common endpoint, cut-offs and timepoints differed across cohorts. Furthermore, although some studies controlled for confounders such as age, body mass index, and NIHSS, others did not include multivariable models, limiting the ability to infer independent associations. The review was not preregistered in PROSPERO, which may increase the risk of selective reporting. We restricted eligibility to acute ischemic stroke to avoid over-generalizing prognostic associations to ICH, SAH, or chronic subdural hematoma; consequently, findings should not be assumed to apply to those populations without dedicated data. Risk-of-bias assessment used ROBINS-I and NOS rather than a prognostic-factor-specific tool such as QUIPS, which may incompletely capture biases unique to prognostic research. Other limitations include exclusive focus on temporalis thickness; temporalis fat-pad thickness (absolute or relative to muscle) and external carotid/STA inflow quality or caliber—features potentially available on MRI or vascular imaging—were not reported in the included studies and warrant future investigation as complementary sarcopenia or perfusion-related markers. Finally, none of the included AIS cohorts directly validated TMT against DXA or consensus EWGSOP/AWGS sarcopenia criteria within the same sample.

Despite these limitations, the collected data link lower TMT to worse neurologic outcomes and higher mortality after acute ischemic stroke. Future prospective studies should standardize TMT acquisition and thresholds, compare TMT with temporal fat-pad and STA-related vascular measures, and test whether integrating TMT into prognostic algorithms or care pathways improves outcomes.

## 5. Conclusions

Taken together, these data demonstrate a consistently observed association in acute ischemic stroke: patients with lower temporalis muscle thickness, defined as sarcopenia by sex-specific thresholds or lower quartiles, experience less functional independence, higher mortality, and more persistent dysphagia, whereas those with higher TMT have better odds of favorable neurological recovery. TMT can be measured quickly from routine imaging and may offer insight into patients’ physiological reserve. However, present evidence remains observational and does not establish that TMT-guided clinical decisions improve outcomes; TMT should therefore be considered a promising prognostic marker requiring prospective validation and standardized measurement rather than a tool already ready for routine decision-making.

Across cohorts, lower TMT is consistently associated with reduced functional recovery after acute ischemic stroke, while higher TMT, whether modelled as a continuous variable or grouped into quartiles, correlates with improved mRS outcomes in individual studies. The high prevalence of TMT-defined sarcopenia in ischemic stroke cohorts raises the possibility that muscle health contributes to vulnerability and recovery, although the observational designs of the included studies do not establish causality. Future studies should examine the long-term impact of sarcopenia, incorporate pre- and post-event TMT (and potentially temporal fat-pad and STA-related measures), and define standardized acquisition and thresholding protocols before TMT is integrated systematically into clinical prognostication.

## Figures and Tables

**Table 1:** Overview of included studies: Summary of included studies evaluating temporalis muscle thickness in acute ischemic stroke.

Study Author	Year	Study Type	Number of Patients	Age (Years)	Sex	Population/Diagnosis	Treatment	Hemoglobin (Mean)	TMT	mRS (Y/N)	Sarcopenia
Yang et al. [17]	2023	Retrospective	148	73.1	M: 68 F: 80	Large vessel occlusion stroke	Thrombolysis	13.1 ± 2.1 g/dL	5.9 mm	Yes	N/A
Ravera et al. [18]	2024	Retrospective	291	73	M:158 F: 133	Acute ischemic stroke	Thrombolysis & Thrombectomy	N/A	5.2 mm	Yes	197 (68%)
Lin et al. [16]	2023	Retrospective	657	72	M: 342 F:315	Large vessel occlusion stroke	Thrombolysis & Thrombectomy	13.2 ± 3.0 g/dL	6.35 mm	Yes	N/A
Li et al. [19]	2022	Retrospective	265	58.5	M: 166 F: 99	Acute ischemic stroke	N/A	13.9 ± 1.73 g/dL	5.3 mm	Yes	131 (49.4%)

Abbreviations: M = male, F = female, N/A = not available, TMT = temporalis muscle thickness, mRS = modified Rankin Scale, Y/N = Yes, No.

**Table 2:** Summary of patient demographics and interventions: Baseline demographics and key clinical characteristics of patients in the included acute ischemic stroke cohorts.

Characteristics (No. of Patients for Whom Information Is Available)	Value (Among Patients with Available Data)
Cohort size (no.)	1361
Mean Patient Age (n = 1361)	69.7 Years
Gender (n = 1361)	No. (%)
Male	734 (53.9%)
Female	627 (46.1%)
Comorbidities (as reported in source cohorts)	No. (%)
Smoking; Diabetes mellitus	Not re-pooled (heterogeneous reporting across AIS cohorts)
Mean Temporalis Muscle Thickness	Study-level means 5.2–6.35 mm (approx. weighted mean of means 5.9 mm; n = 1361)
Sarcopenia Status (n = 704 with reported status)	No. (%)
Sarcopenic	365 (51.8%)
Study-level mean temporalis muscle thickness ± SD	Not uniformly reported across all AIS cohorts
Non-Sarcopenic	339 (48.2%)
Study-level mean temporalis muscle thickness ± SD	Not uniformly reported across all AIS cohorts
Intervention (among cohorts reporting reperfusion therapy)	See source AIS cohorts (Yang, Ravera, Lin); Li did not uniformly report reperfusion fractions
Thrombectomy	Reported in EVT/thrombolysis AIS cohorts (not formally re-pooled)
Thrombolysis	Reported in EVT/thrombolysis AIS cohorts (not formally re-pooled)
Modified Rankin Score (descriptive; not a formal pooled estimate) *	Reported by study in Table 3; overall category % not formally pooled
0–2 (functional independence)	See study-level results
3–4 (moderate to moderately severe disability)	See study-level results
5–6 (severe disability and death)	See study-level results

* mRS categories are descriptive summaries from cohorts reporting overall functional outcomes and should not be interpreted as formal meta-analytic pooled estimates. Sarcopenia-stratified mRS distributions were not uniformly reported across studies and are therefore described narratively and by study in Table 3 rather than aggregated as a single pooled effect.

**Table 3:** Patient outcomes: Definitions of sarcopenia by temporalis muscle thickness and reporting of modified Rankin Scale outcomes across included acute ischemic stroke studies.

Study	n=	Sarcopenia (%)	Key Outcome Findings (mRS and Survival)
Yang [17]	148	37 (25.0%)	Primary outcome: NG tube dependence; no mRS reported
Ravera [18]	291	197 (68.0%)	mRS of 0–1 assoc. with higher TMT (avg = 5.5 mm)
Lin [16]	657	Not reported	mRS of ≤2 associated with the highest TMT group (n = 141, TMT = 8.82 mm)
Li [19]	265	131 (49.4%)	mRS 0–2: 80/131 (61%) in sarcopenic; 90/134 (67%) in non-sarcopenic

## Data Availability

No new data were created or analyzed in this study. Data sharing is not applicable to this article.

## Supplementary Materials

The following supporting information can be downloaded at https://www.mdpi.com/article/10.3390/jcm15187240/s1, Table S1: Full database-specific search strategies for PubMed, MEDLINE (Ovid), Embase, Web of Science, and Scopus; Table S2: PRISMA 2020 Checklist.
