# Supplementary material for: Temporalis Muscle Thickness as a Prognostic Marker in Acute Ischemic Stroke: A Systematic Review
Source: J Clin Med. 2026 Sep 17;15(18):7240. doi: 10.3390/jcm15187240 (PMC13607217; doi:10.3390/jcm15187240)
Supplement: Supplementary file 1 [file jcm-15-07240-s001.zip › Supplementary Table S1.pdf]

Table S1: Full database-specific search strategies for PubMed, MEDLINE (Ovid), Embase, Web of Science, and Scopus.

Database	Search strategy	Limits
**PubMed**	("temporalis muscle thickness"[Title/Abstract] OR "temporal muscle thickness"[Title/Abstract] OR ("temporalis muscle"[Title/Abstract] AND thickness[Title/Abstract]) OR "temporal muscle"[Title/Abstract]) AND (stroke[Title/Abstract] OR "cerebrovascular accident"[Title/Abstract] OR "ischemic stroke"[Title/Abstract] OR "ischaemic stroke"[Title/Abstract] OR "intracerebral hemorrhage"[Title/Abstract] OR "cerebrovascular disease"[Title/Abstract] OR "chronic subdural hematoma"[Title/Abstract] OR "chronic subdural haematoma"[Title/Abstract]) AND (sarcopenia[Title/Abstract] OR "muscle mass"[Title/Abstract] OR "muscle wasting"[Title/Abstract] OR frailty[Title/Abstract] OR outcome*[Title/Abstract])	Publication date: 2000 to December 2025; Humans; Adults
**MEDLINE (Ovid)**	1. temporalis muscle.tw,kf. OR temporal muscle.tw,kf. 2. exp Sarcopenia/ OR sarcopenia.tw,kf. OR muscle mass.tw,kf. OR muscle wasting.tw,kf. 3. exp Stroke/ OR exp Cerebrovascular Disorders/ OR stroke.tw,kf. OR cerebrovascular accident*.tw,kf. OR intracerebral hemorrhage.tw,kf. OR chronic subdural hematoma.tw,kf. OR chronic subdural haematoma.tw,kf. 4. outcome*.tw,kf. 5. 1 AND 2 AND 3 AND 4	Limited to humans; years = 2000–2025 (search run through December 2025)
**Embase (Elsevier)**	1. 'temporal muscle'/exp OR 'temporalis muscle':ti,ab OR 'temporal muscle':ti,ab 2. 'sarcopenia'/exp OR sarcopenia:ti,ab OR 'muscle mass':ti,ab OR 'muscle wasting':ti,ab 3. 'stroke'/exp OR 'cerebrovascular disease'/exp OR stroke:ti,ab OR 'cerebrovascular accident*':ti,ab OR 'intracerebral hemorrhage':ti,ab OR 'chronic subdural hematoma':ti,ab OR 'chronic subdural	Years: 2000–2025; Humans; Adults (search run through December 2025)

	haematoma':ti,ab 4. outcome*:ti,ab 5. 1 AND 2 AND 3 AND 4	
**Web of Science (Core Collection)**	TS=("temporalis muscle thickness" OR "temporal muscle thickness" OR ("temporalis muscle" AND thickness) OR "temporal muscle") AND TS=(stroke OR "cerebrovascular accident" OR "ischemic stroke" OR "ischaemic stroke" OR "intracerebral hemorrhage" OR "cerebrovascular disease" OR "chronic subdural hematoma" OR "chronic subdural haematoma") AND TS=(sarcopenia OR "muscle mass" OR "muscle wasting" OR frailty OR outcome*)	Timespan: 2000–December 2025; Document types: Article, Review; Language: English
**Scopus**	TITLE-ABS-KEY("temporalis muscle thickness" OR "temporal muscle thickness" OR ("temporalis muscle" AND thickness) OR "temporal muscle") AND TITLE-ABS-KEY(stroke OR "cerebrovascular accident" OR "ischemic stroke" OR "ischaemic stroke" OR "intracerebral hemorrhage" OR "cerebrovascular disease" OR "chronic subdural hematoma" OR "chronic subdural haematoma") AND TITLE-ABS-KEY(sarcopenia OR "muscle mass" OR "muscle wasting" OR frailty OR outcome*)	Years: 2000–2025; Document type: Article, Review; Humans; Adults
