# Supplementary material for: Temporalis Muscle Thickness as a Prognostic Marker in Acute Ischemic Stroke: A Systematic Review
Source: J Clin Med. 2026 Sep 17;15(18):7240. doi: 10.3390/jcm15187240 (PMC13607217; doi:10.3390/jcm15187240)
Supplement: Supplementary file 1 [file jcm-15-07240-s001.zip › Supplementary Table S2-PRISMA_Checklist.pdf]

PRISMA 2020 Checklist

Section and Topic	Item #	Checklist item	Location where item is reported
**TITLE**			
Title- Temporalis Muscle Thickness as a Prognostic Marker in Acute Ischemic Stroke: A Systematic Review	1	Systematic Review	Title page
**ABSTRACT**			
Abstract	2	See the PRISMA 2020 for Abstracts checklist.	Abstract
**INTRODUCTION**			
Rationale	3	Paragraphs 1–3: stroke burden, sarcopenia, rationale for TMT as a neuroimaging-derived prognostic marker, and evidence gap in acute ischemic stroke.	Introduction
Objectives	4	To assess whether TMT is associated with functional status, dysphagia, and mortality in adults with acute ischemic stroke.	Last paragraph of intro
**METHODS**			
Eligibility criteria	5	Methods, Section 2.2, Study Selection: adults with acute ischemic stroke, TMT measurement, and at least one clinical outcome with follow-up. Excluded populations were hemorrhagic stroke alone, mixed cerebrovascular cohorts without AIS-specific analysis, chronic subdural hematoma, studies without TMT measurement, and studies without outcome or follow-up data.	Methods
Information sources	6	Methods, Section 2.1, Literature Review: PubMed, MEDLINE via Ovid, Embase, Web of Science, and Scopus, searched from 2000 through December 2025.	Methods
Search strategy	7	Methods, Section 2.1, Literature Review; Supplementary Table S1 provides complete database-specific search strategies, Boolean operators, and applied limits.	Methods
Selection process	8	Methods, Sections 2.2 and 2.3: two reviewers independently screened titles/abstracts and assessed full texts; a third reviewer resolved disagreements.	Methods
Data collection process	9	Methods, Section 2.3, Data Extraction: two reviewers independently extracted data from included studies; disagreements were resolved by a third reviewer.	Methods
Data items	10a	Methods, Sections 2.3–2.4; Results, Sections 3.2–3.5: functional outcomes including mRS, mortality/survival, dysphagia or nasogastric tube dependence, and systemic muscle-mass associations where reported.	Methods
	10b	Methods, Section 2.3; Results, Section 3.1 and Tables 1–3: study design, sample size, age, sex, stroke population, intervention/reperfusion therapy, TMT measurement, TMT-defined sarcopenia, and outcome time points.	Methods
Study risk of bias assessment	11	Methods, Section 2.5, Risk of Bias/Quality of Evidence: two reviewers assessed risk of bias. ROBINS-I was applied to Yang, Ravera, and Lin; the Newcastle–Ottawa Scale was applied to Li. The rationale for these tools and the limitation relative to QUIPS are described.	Methods
Effect measures	12	Methods, Section 2.6, Statistical Analysis; Results, Sections 3.2–3.4: no meta-analysis was performed. Adjusted odds ratios, hazard ratios, AUCs, and other effect estimates are reported narratively as presented in the individual studies.	Methods
Synthesis methods	13a	Methods, Sections 2.4 and 2.6: narrative synthesis was used because of heterogeneity in TMT definitions, sarcopenia thresholds, outcome measures, mRS cutoffs, and outcome time points.	Methods
	13b	Not applicable: no quantitative meta-analysis or transformation of effect estimates was performed. Study-level findings were extracted and	N/A

Section and Topic	Item #	Checklist item	Location where item is reported
		narratively summarized.	
	13c	Results, Tables 1–3 and Figures 2–3: study characteristics, patient demographics, sarcopenia definitions, functional outcomes, and risk-of-bias assessments are presented by study.	Results
	13d	Methods, Section 2.6; Results, Sections 3.2–3.5: findings were synthesized narratively by outcome domain. Formal pooling was not performed because of clinical and methodological heterogeneity.	Methods
	13e	Not applicable: no quantitative heterogeneity analyses or subgroup analyses were undertaken. Sources of heterogeneity are described narratively in Methods, Results, and Limitations.	N/A
	13f	Not applicable: no sensitivity analyses were performed because no meta-analysis was conducted.	N/A
Reporting bias assessment	14	Not formally assessed because fewer than 10 studies were included and no meta-analysis was performed; the potential for publication/reporting bias is acknowledged in Section 4.5, Limitations.	Limitations
Certainty assessment	15	Not formally assessed; certainty of evidence was not graded. This is acknowledged in Section 4.5, Limitations.	Limitations
**RESULTS**			
Study selection	16a	Results, Section 3.1, Literature Search and Study Characteristics; Figure 1: 42 records identified, 15 duplicates removed, 27 records screened, 19 records excluded, eight full-text reports assessed, four full-text reports excluded, and four studies included.	Results
	16b	Figure 1; Results, Section 3.1: four full-text reports were excluded because the population was not restricted to acute ischemic stroke.	Figure 1
Study characteristics	17	Results, Section 3.1; Tables 1–3: four acute ischemic stroke cohorts comprising 1,361 patients are summarized.	Results
Risk of bias in studies	18	Results, Section 3.6, Risk of Bias; Figures 2–3: ROBINS-I findings are reported for Yang, Ravera, and Lin, and Newcastle–Ottawa Scale findings for Li.	Results
Results of individual studies	19	Results, Sections 3.2–3.5; Tables 1–3: study-level results for functional outcome, mortality/survival, dysphagia/NG-tube dependence, and systemic muscle mass are presented narratively and in tables.	Results
Results of syntheses	20a	Results, Sections 3.1–3.6: characteristics, risk of bias, and outcome-specific findings are summarized narratively.	Results
	20b	Not applicable (no meta-analysis). Not performed; narrative synthesis only.	N/A
	20c	Not applicable: no formal heterogeneity analyses were conducted; heterogeneity is described qualitatively in Results and Limitations.	N/A
	20d	Not applicable (no meta-analysis). Not performed; narrative synthesis only.	N/A
Reporting biases	21	Not formally assessed; the potential for publication/reporting bias is acknowledged in Section 4.5, Limitations.	Limitations
Certainty of evidence	22	Not formally assessed; certainty of evidence was not graded and is acknowledged in Section 4.5, Limitations.	Limitations
**DISCUSSION**			
Discussion	23a	Discussion, Section 4.1, Summary of Key Findings.	Discussion
	23b	Discussion, Section 4.5, Limitations: heterogeneous TMT thresholds and sarcopenia definitions, variable mRS cutoffs and time points, observational designs, incomplete adjustment for confounders, lack of AIS-specific validation against DXA/bioimpedance or consensus sarcopenia criteria, and no evidence that TMT-guided management improves outcomes.	Discussion
	23c	Discussion, Section 4.5, Limitations: review not prospectively registered; no protocol was prepared; reporting/publication bias was not formally assessed; and ROBINS-I/NOS rather than QUIPS were used for prognostic-factor studies.	Limitations
	23d	Discussion, Section 4.4, Implications for Practice and Imaging-Informed Rehabilitation; Conclusion: TMT is a promising imaging-derived	Discussion

PRISMA 2020 Checklist

Section and Topic	Item #	Checklist item	Location where item is reported
		prognostic marker that requires prospective validation and standardized measurement before routine clinical decision-making.	
**OTHER INFORMATION**			
Registration and protocol	24a	Methods, Section 2.1; Discussion, Section 4.5: the review was not prospectively registered in PROSPERO.	Methods
	24b	No protocol prepared.	N/A
	24c	Not applicable	N/A
Support	25	This research received no specific grant from any funding agency in the public, commercial or not-for-profit sectors.	Funding statement
Competing interests	26	The authors declare no competing interests.	Competing interests
Availability of data, code and other materials	27	Not applicable; analysis based on published aggregate data only; no additional datasets or code generated; all data analyzed were extracted from published studies and are reported in the article and its supplementary materials; no new datasets or code were generated.	

From: Page MJ, McKenzie JE, Bossuyt PM, Boutron I, Hoffmann TC, Mulrow CD, et al. The PRISMA 2020 statement: an updated guideline for reporting systematic reviews. BMJ 2021;372:n71. doi: 10.1136/bmj.n71
